# 1,8-Bis[3-(triethoxy­silyl)prop­yl]-1,8-diazo­niatricyclo­[9.3.1.1^4,8^]hexa­decane diiodide

**DOI:** 10.1107/S1600536809038112

**Published:** 2009-09-26

**Authors:** Yoann Rousselin, Franck Denat, Benedicte Lebeau, Alain Walcarius

**Affiliations:** a ICMUB, UMR5260, CNRS–Université de Bourgogne, 9 Avenue Alain Savary, 21078 Dijon Cedex, France; b LMPC, UMR 7016, CNRS–ENSCMu–UHA, 3 Rue A. Werner, 68093 Mulhouse cedex, France; c LCPME, UMR 7564, CNRS–Nancy Université, 405 Rue de Vandoeuvre, 54600 Villers-les-Nancy, France

## Abstract

The organic mol­ecule of title compound, C_30_H_66_N_4_O_6_Si_2_^2+^·2I^−^, is located around a centre of symmetry. The structure exhibits disorder of the trieth­oxy groups with the ratios 0.78 (1)/0.22 (1), 0.67 (1)/0.33 (1) and 0.58 (1)/0.42 (1).

## Related literature

For Si—O bond distances, see: Klapdohr *et al.* (2000 ▶); Bedford *et al.* (2001 ▶); Aksin *et al.* (2006 ▶).
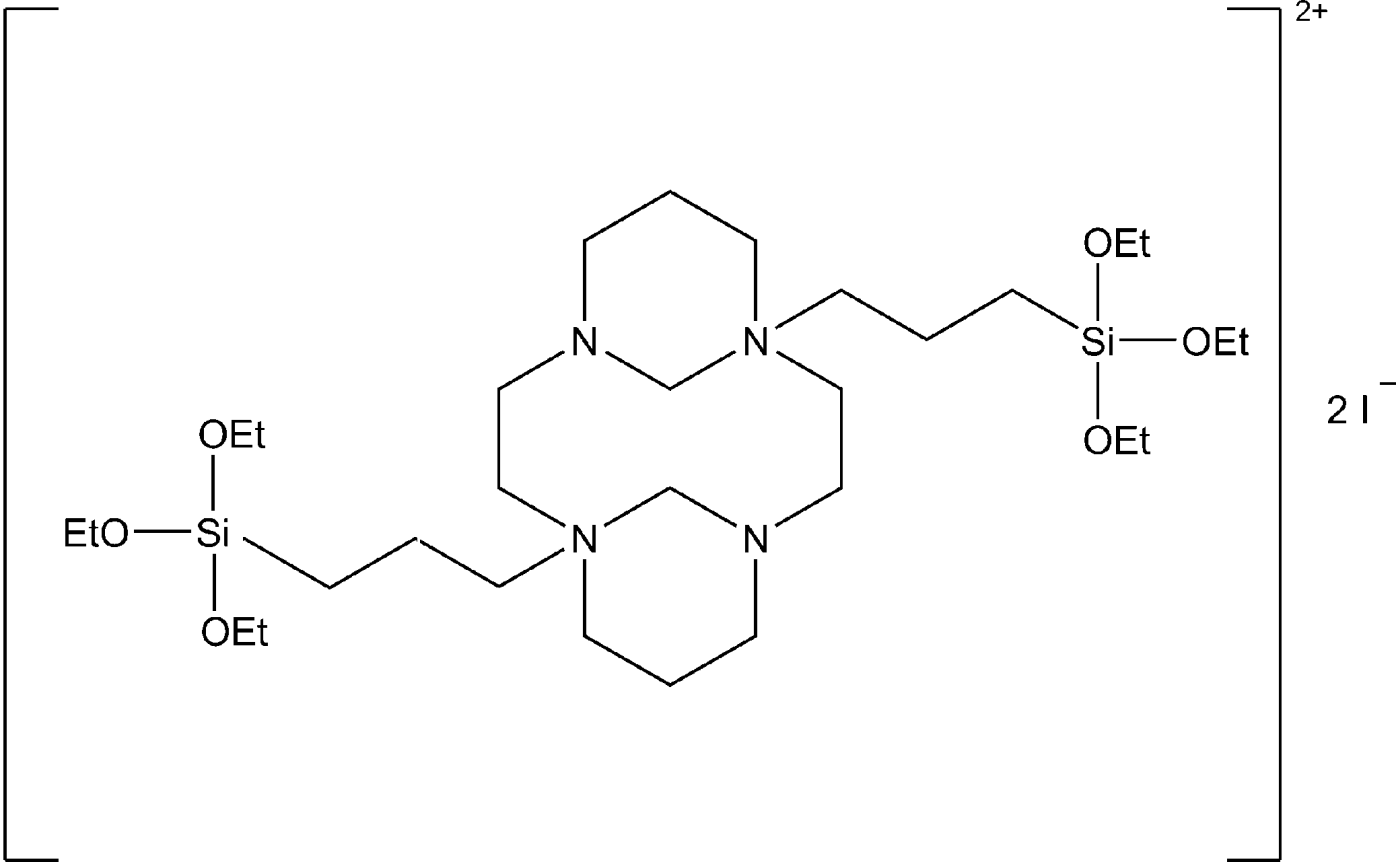


## Experimental

### 

#### Crystal data

C_30_H_66_N_4_O_6_Si_2_^2+^·2I^−^
*M* *_r_* = 888.85
Monoclinic, 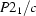

*a* = 15.0484 (2) Å
*b* = 8.4229 (1) Å
*c* = 16.5921 (3) Å
β = 101.808 (1)°
*V* = 2058.57 (5) Å^3^
*Z* = 2
Mo *K*α radiation
μ = 1.63 mm^−1^
*T* = 115 K
0.21 × 0.21 × 0.15 mm

#### Data collection

Nonius Kappa CCD diffractometer
Absorption correction: none
8944 measured reflections
4686 independent reflections
3900 reflections with *I* > 2σ(*I*)
*R*_int_ = 0.030

#### Refinement

*R*[*F* ^2^ > 2σ(*F* ^2^)] = 0.030
*wR*(*F* ^2^) = 0.076
*S* = 1.03
4686 reflections
208 parameters
17 restraints
H-atom parameters constrained
Δρ_max_ = 0.80 e Å^−3^
Δρ_min_ = −0.84 e Å^−3^

### 

Data collection: *COLLECT* (Nonius, 2000 ▶); cell refinement: *SCALEPACK* (Otwinowski & Minor, 1997 ▶); data reduction: *DENZO* (Otwinowski & Minor, 1997 ▶) and *SCALEPACK*; program(s) used to solve structure: *SIR92* (Altomare *et al.*, 1993 ▶); program(s) used to refine structure: *SHELXL97* (Sheldrick, 2008 ▶); molecular graphics: *ORTEP-3* (Farrugia, 1997 ▶); software used to prepare material for publication: *WinGX* (Farrugia, 1999 ▶).

## Supplementary Material

Crystal structure: contains datablocks I, global. DOI: 10.1107/S1600536809038112/rk2168sup1.cif

Structure factors: contains datablocks I. DOI: 10.1107/S1600536809038112/rk2168Isup2.hkl

Additional supplementary materials:  crystallographic information; 3D view; checkCIF report

## Figures and Tables

**Fig. 1.**
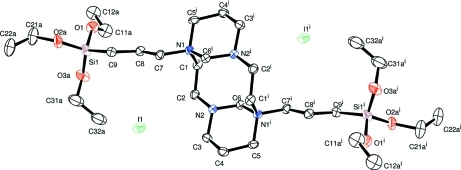
Molecular view of I with the atom numbering scheme. Displacement ellipsoids are drawn at the 50% probability level. H atoms are omitted for clarity. Only major molecular moieties for disordered parts are shown. Symmetry codes: (i) -x+1, -y, -z.

## supplementary crystallographic information

### Comment

The molecule of title compound, C_30_H_66_N_4_O_6_Si_2_^.^2(I), placed around of centre of symmetry. The Si—O bond distances (Si1—O1 = 1.615 (2), Si1—O2B = 1.629 (7), Si1—O2A = 1.633 (4), Si1—O3A = 1.635 (5), Si1—O3B = 1.644 (6) Å) are in good agreement with those observed in literature - Klapdohr *et al.*, (2000); Bedford *et al.*, (2001); Aksin *et al.*, (2006) - Si—O = 1.611 (10)–1.644 (12)Å. The molecular structure shows a *trans*–conformation for the two methylenic bridge (Fig. 1).

### Experimental

The 59.5 g of iodopropyltriethoxysilane (2 eq., 0.18 mol) were added to a solution of formaldehyde–cyclam (purchased from CheMatech) (20.07 g, 0.09 mol) in freshly distilled acetonitrile (180 ml) under N_2_. The white precipitate formed after 3 h was filtered, rinsed with acetonitrile (50 ml). The resulting solid was dried under vacuum. The title compound, I, was obtained as a white solid (m = 42.74 g, 46.5 mmol, yield = 51.6%). No trace of *cis*–disubstituted macrocycle was detected, indicating a strong selectivity for the *trans*–disubstitution. Crystals of I suitable for single–crystal *X*–ray diffraction were selected directly from the sample.

^13^C [^1^H] NMR (75 MHz, CDCl_3_, 300 K): (CH_2_) 7.2, 15.2, (CH_3_) 18.7, (CH_2_) 19.6, 46.8, 46.9, 51.3, (*O–*CH_2_) 58.4, (CH_2_) 59.2, 60.8, 77.0. MALDI–TOF: m/*z* = 635.02 [*M*^+^].

### Refinement

All H atoms were placed at calculated position using a riding model with C—H = 0.98Å (methyl) or 0.99Å (methylene) with *U*_iso_(H) = 1.2*U*_eq_(CH_2_) or *U*_iso_(H) = 1.5*U*_eq_(CH_3_).

Two of the triethoxy groups and one ethane group are disordered over two positions. The geometric parameters of three disordered components in each groups were restrained by using SADI restraints and using EADP constraints (Sheldrick, 2008). In the final stages of refinement, the statistical fractions of the major and minor disordered components were held fixed to the nearest rounded values of 0.78/0.22, 0.67/0.33 and 0.58/0.42 for respectively each disordered group. Similar *U^ij^* constraints were applied within the disordered parts to maintain a reasonable model. However, these disordered parts of the molecule display rather large ellipsoids with respect to the central core resulting in an Alert C on a Large Non–Solvent H or C *U*_eq_(max)/*U*_eq_(min) ratio.

### Crystal data

**Table:**

C_30_H_66_N_4_O_6_Si_2_^2+^·2I^−^	*F*(000) = 912
*M**_r_* = 888.85	*D*_x_ = 1.434 Mg m^−^^3^
Monoclinic, *P*2_1_/*c*	Mo *K*α radiation, λ = 0.71073 Å
Hall symbol: -P 2ybc	Cell parameters from 4978 reflections
*a* = 15.0484 (2) Å	θ = 1.0–27.5°
*b* = 8.4229 (1) Å	µ = 1.63 mm^−^^1^
*c* = 16.5921 (3) Å	*T* = 115 K
β = 101.808 (1)°	Prism, colourless
*V* = 2058.57 (5) Å^3^	0.21 × 0.21 × 0.15 mm
*Z* = 2	

### Data collection

**Table:**

Nonius Kappa CCD diffractometer	3900 reflections with *I* > 2σ(*I*)
Radiation source: fine–focus sealed tube	*R*_int_ = 0.030
graphite	θ_max_ = 27.5°, θ_min_ = 2.9°
Detector resolution: 9 pixels mm^-1^	*h* = −19→19
φ and ω scans	*k* = −10→10
8944 measured reflections	*l* = −21→21
4686 independent reflections	

### Refinement

**Table:**

Refinement on *F*^2^	Primary atom site location: structure-invariant direct methods
Least-squares matrix: full	Secondary atom site location: difference Fourier map
*R*[*F*^2^ > 2σ(*F*^2^)] = 0.030	Hydrogen site location: inferred from neighbouring sites
*wR*(*F*^2^) = 0.076	H-atom parameters constrained
*S* = 1.03	*w* = 1/[σ^2^(*F*_o_^2^) + (0.0349*P*)^2^ + 1.6474*P*] where *P* = (*F*_o_^2^ + 2*F*_c_^2^)/3
4686 reflections	(Δ/σ)_max_ = 0.002
208 parameters	Δρ_max_ = 0.80 e Å^−^^3^
17 restraints	Δρ_min_ = −0.84 e Å^−^^3^
16 constraints	

### Special details

**Table:**

Geometry. All s.u.'s (except the s.u. in the dihedral angle between two l.s. planes) are estimated using the full covariance matrix. The cell s.u.'s are taken into account individually in the estimation of s.u.'s in distances, angles and torsion angles; correlations between s.u.'s in cell parameters are only used when they are defined by crystal symmetry. An approximate (isotropic) treatment of cell s.u.'s is used for estimating s.u.'s involving l.s. planes.
Refinement. Refinement of *F*^2^ against ALL reflections. The weighted *R*–factor *wR* and goodness of fit *S* are based on *F*^2^, conventional *R*–factors *R* are based on *F*, with *F* set to zero for negative *F*^2^. The threshold expression of *F*^2^ > σ(*F*^2^) is used only for calculating *R*–factors(gt) *etc*. and is not relevant to the choice of reflections for refinement. *R*–factors based on *F*^2^ are statistically about twice as large as those based on *F*, and *R*–factors based on ALL data will be even larger.

### Fractional atomic coordinates and isotropic or equivalent isotropic displacement parameters (Å^2^)

**Table:**

	*x*	*y*	*z*	*U*_iso_*/*U*_eq_	Occ. (<1)
C1	0.59893 (17)	0.0563 (3)	0.11258 (14)	0.0219 (5)	
H1A	0.5638	−0.0392	0.1215	0.026*	
H1B	0.6450	0.0741	0.1636	0.026*	
C2	0.64831 (17)	0.0206 (3)	0.04340 (14)	0.0231 (5)	
H2A	0.6804	0.1179	0.0318	0.028*	
H2B	0.6949	−0.0616	0.0627	0.028*	
C3	0.64235 (18)	−0.0542 (3)	−0.09955 (16)	0.0275 (6)	
H3A	0.6837	−0.1465	−0.0873	0.033*	
H3B	0.6793	0.0417	−0.1032	0.033*	
C4	0.5753 (2)	−0.0802 (3)	−0.18030 (15)	0.0289 (6)	
H4A	0.5414	0.0195	−0.1962	0.035*	
H4B	0.6093	−0.1057	−0.2238	0.035*	
C5	0.50826 (18)	−0.2128 (3)	−0.17557 (14)	0.0244 (5)	
H5A	0.5400	−0.3162	−0.1734	0.029*	
H5B	0.4599	−0.2113	−0.2260	0.029*	
C6	0.54212 (16)	−0.1799 (3)	−0.02599 (13)	0.0192 (5)	
H6A	0.5175	−0.1772	0.0250	0.023*	
H6B	0.5839	−0.2715	−0.0227	0.023*	
C7	0.58228 (17)	0.3514 (3)	0.08780 (14)	0.0214 (5)	
H7A	0.5970	0.3490	0.0323	0.026*	
H7B	0.5389	0.4396	0.0884	0.026*	
C8	0.66860 (17)	0.3891 (3)	0.14980 (15)	0.0232 (5)	
H8A	0.7175	0.3148	0.1428	0.028*	
H8B	0.6581	0.3774	0.2065	0.028*	
C9	0.69656 (17)	0.5607 (3)	0.13546 (15)	0.0241 (5)	
H9A	0.6483	0.6334	0.1457	0.029*	
H9B	0.7009	0.5726	0.0770	0.029*	
Si1	0.80597 (5)	0.62166 (9)	0.20138 (5)	0.02915 (17)	
O1	0.81232 (14)	0.5729 (2)	0.29651 (11)	0.0361 (5)	
C11A	0.8556 (3)	0.4338 (5)	0.3373 (2)	0.0469 (7)	0.78
H11A	0.9028	0.3950	0.3084	0.056*	0.78
H11B	0.8103	0.3484	0.3366	0.056*	0.78
C12A	0.8981 (3)	0.4762 (6)	0.4245 (2)	0.0469 (7)	0.78
H12A	0.9275	0.3821	0.4530	0.070*	0.78
H12B	0.8510	0.5143	0.4528	0.070*	0.78
H12C	0.9434	0.5598	0.4248	0.070*	0.78
O2A	0.8055 (5)	0.8155 (4)	0.1990 (3)	0.0331 (13)	0.67
C21A	0.8750 (5)	0.9184 (6)	0.2443 (4)	0.0513 (14)	0.67
H21A	0.9327	0.8592	0.2608	0.062*	0.67
H21B	0.8567	0.9577	0.2947	0.062*	0.67
C22A	0.8879 (5)	1.0565 (6)	0.1897 (4)	0.0513 (14)	0.67
H22A	0.9363	1.1256	0.2190	0.077*	0.67
H22B	0.8312	1.1169	0.1754	0.077*	0.67
H22C	0.9045	1.0165	0.1393	0.077*	0.67
O3A	0.8982 (4)	0.5450 (14)	0.1800 (4)	0.0372 (16)	0.58
C31A	0.9148 (5)	0.5705 (8)	0.0983 (4)	0.0554 (10)	0.58
H31A	0.9762	0.6158	0.1020	0.066*	0.58
H31B	0.8699	0.6470	0.0683	0.066*	0.58
C32A	0.9074 (5)	0.4163 (7)	0.0524 (4)	0.0554 (10)	0.58
H32A	0.9235	0.4327	−0.0013	0.083*	0.58
H32B	0.8450	0.3767	0.0444	0.083*	0.58
H32C	0.9489	0.3386	0.0841	0.083*	0.58
C11B	0.8814 (9)	0.5688 (15)	0.3700 (7)	0.0469 (7)	0.22
H11C	0.8631	0.6319	0.4143	0.056*	0.22
H11D	0.9394	0.6111	0.3596	0.056*	0.22
C12B	0.8901 (12)	0.3975 (16)	0.3927 (10)	0.0469 (7)	0.22
H12D	0.9334	0.3855	0.4452	0.070*	0.22
H12E	0.9121	0.3384	0.3497	0.070*	0.22
H12F	0.8308	0.3560	0.3981	0.070*	0.22
O2B	0.8147 (12)	0.8091 (9)	0.1799 (8)	0.0331 (13)	0.33
C21B	0.8921 (11)	0.8929 (11)	0.2268 (11)	0.0513 (14)	0.33
H21C	0.9471	0.8657	0.2056	0.062*	0.33
H21D	0.9022	0.8599	0.2853	0.062*	0.33
C22B	0.8762 (11)	1.0688 (11)	0.2205 (8)	0.0513 (14)	0.33
H22D	0.9308	1.1245	0.2490	0.077*	0.33
H22E	0.8250	1.0965	0.2459	0.077*	0.33
H22F	0.8625	1.1002	0.1624	0.077*	0.33
O3B	0.8823 (7)	0.521 (2)	0.1642 (6)	0.0372 (16)	0.42
C31B	0.8901 (7)	0.4696 (11)	0.0824 (5)	0.0554 (10)	0.42
H31C	0.8350	0.4095	0.0568	0.066*	0.42
H31D	0.9431	0.3982	0.0866	0.066*	0.42
C32B	0.9010 (6)	0.6094 (10)	0.0298 (5)	0.0554 (10)	0.42
H32D	0.9007	0.5734	−0.0264	0.083*	0.42
H32E	0.9588	0.6625	0.0522	0.083*	0.42
H32F	0.8509	0.6839	0.0291	0.083*	0.42
N1	0.53482 (13)	0.1971 (2)	0.10041 (11)	0.0190 (4)	
N2	0.59027 (13)	−0.0345 (3)	−0.03396 (11)	0.0205 (4)	
I1	0.660451 (12)	0.40929 (2)	−0.121474 (10)	0.03066 (7)	

### Atomic displacement parameters (Å^2^)

**Table:**

	*U*^11^	*U*^22^	*U*^33^	*U*^12^	*U*^13^	*U*^23^
C1	0.0257 (13)	0.0179 (12)	0.0204 (11)	0.0039 (10)	0.0006 (9)	0.0014 (9)
C2	0.0217 (12)	0.0210 (13)	0.0261 (12)	0.0012 (10)	0.0039 (10)	−0.0022 (10)
C3	0.0302 (14)	0.0256 (14)	0.0310 (13)	−0.0024 (11)	0.0161 (11)	−0.0011 (11)
C4	0.0406 (16)	0.0270 (14)	0.0229 (12)	−0.0020 (12)	0.0151 (11)	0.0014 (10)
C5	0.0343 (14)	0.0242 (13)	0.0164 (11)	0.0024 (11)	0.0094 (10)	−0.0012 (10)
C6	0.0202 (12)	0.0190 (12)	0.0178 (10)	0.0016 (9)	0.0027 (9)	0.0005 (9)
C7	0.0270 (13)	0.0158 (11)	0.0208 (11)	−0.0001 (10)	0.0035 (9)	0.0001 (9)
C8	0.0238 (13)	0.0221 (13)	0.0232 (12)	0.0016 (10)	0.0038 (10)	−0.0015 (10)
C9	0.0248 (13)	0.0246 (13)	0.0233 (12)	−0.0007 (10)	0.0062 (10)	−0.0002 (10)
Si1	0.0226 (4)	0.0339 (4)	0.0295 (4)	−0.0054 (3)	0.0018 (3)	0.0045 (3)
O1	0.0393 (12)	0.0396 (12)	0.0270 (10)	0.0019 (9)	0.0011 (8)	0.0013 (8)
C11A	0.0436 (16)	0.056 (2)	0.0373 (16)	0.0062 (15)	−0.0013 (13)	0.0084 (13)
C12A	0.0436 (16)	0.056 (2)	0.0373 (16)	0.0062 (15)	−0.0013 (13)	0.0084 (13)
O2A	0.036 (2)	0.0343 (12)	0.026 (3)	−0.0153 (11)	−0.001 (2)	0.0030 (13)
C21A	0.044 (3)	0.0352 (16)	0.066 (3)	−0.0109 (14)	−0.009 (2)	−0.0051 (17)
C22A	0.044 (3)	0.0352 (16)	0.066 (3)	−0.0109 (14)	−0.009 (2)	−0.0051 (17)
O3A	0.013 (2)	0.066 (4)	0.030 (3)	−0.004 (3)	−0.002 (2)	0.002 (2)
C31A	0.047 (2)	0.076 (3)	0.050 (2)	−0.005 (2)	0.0256 (18)	−0.001 (2)
C32A	0.047 (2)	0.076 (3)	0.050 (2)	−0.005 (2)	0.0256 (18)	−0.001 (2)
C11B	0.0436 (16)	0.056 (2)	0.0373 (16)	0.0062 (15)	−0.0013 (13)	0.0084 (13)
C12B	0.0436 (16)	0.056 (2)	0.0373 (16)	0.0062 (15)	−0.0013 (13)	0.0084 (13)
O2B	0.036 (2)	0.0343 (12)	0.026 (3)	−0.0153 (11)	−0.001 (2)	0.0030 (13)
C21B	0.044 (3)	0.0352 (16)	0.066 (3)	−0.0109 (14)	−0.009 (2)	−0.0051 (17)
C22B	0.044 (3)	0.0352 (16)	0.066 (3)	−0.0109 (14)	−0.009 (2)	−0.0051 (17)
O3B	0.013 (2)	0.066 (4)	0.030 (3)	−0.004 (3)	−0.002 (2)	0.002 (2)
C31B	0.047 (2)	0.076 (3)	0.050 (2)	−0.005 (2)	0.0256 (18)	−0.001 (2)
C32B	0.047 (2)	0.076 (3)	0.050 (2)	−0.005 (2)	0.0256 (18)	−0.001 (2)
N1	0.0221 (10)	0.0184 (10)	0.0164 (9)	0.0012 (8)	0.0039 (8)	0.0008 (8)
N2	0.0217 (11)	0.0202 (10)	0.0200 (9)	−0.0016 (8)	0.0051 (8)	−0.0024 (8)
I1	0.03902 (12)	0.02923 (11)	0.02399 (10)	0.00690 (8)	0.00707 (7)	0.00048 (7)

### Geometric parameters (Å, °)

**Table:**

C1—N1	1.516 (3)	C11A—H11B	0.9900
C1—C2	1.520 (3)	C12A—H12A	0.9800
C1—H1A	0.9900	C12A—H12B	0.9800
C1—H1B	0.9900	C12A—H12C	0.9800
C2—N2	1.472 (3)	O2A—C21A	1.446 (5)
C2—H2A	0.9900	C21A—C22A	1.510 (6)
C2—H2B	0.9900	C21A—H21A	0.9900
C3—N2	1.475 (3)	C21A—H21B	0.9900
C3—C4	1.519 (4)	C22A—H22A	0.9800
C3—H3A	0.9900	C22A—H22B	0.9800
C3—H3B	0.9900	C22A—H22C	0.9800
C4—C5	1.518 (4)	O3A—C31A	1.444 (7)
C4—H4A	0.9900	C31A—C32A	1.498 (6)
C4—H4B	0.9900	C31A—H31A	0.9900
C5—N1^i^	1.524 (3)	C31A—H31B	0.9900
C5—H5A	0.9900	C32A—H32A	0.9800
C5—H5B	0.9900	C32A—H32B	0.9800
C6—N2	1.443 (3)	C32A—H32C	0.9800
C6—N1^i^	1.517 (3)	C11B—C12B	1.490 (8)
C6—H6A	0.9900	C11B—H11C	0.9900
C6—H6B	0.9900	C11B—H11D	0.9900
C7—C8	1.516 (3)	C12B—H12D	0.9800
C7—N1	1.518 (3)	C12B—H12E	0.9800
C7—H7A	0.9900	C12B—H12F	0.9800
C7—H7B	0.9900	O2B—C21B	1.446 (7)
C8—C9	1.538 (3)	C21B—C22B	1.501 (8)
C8—H8A	0.9900	C21B—H21C	0.9900
C8—H8B	0.9900	C21B—H21D	0.9900
C9—Si1	1.853 (3)	C22B—H22D	0.9800
C9—H9A	0.9900	C22B—H22E	0.9800
C9—H9B	0.9900	C22B—H22F	0.9800
Si1—O1	1.6148 (19)	O3B—C31B	1.452 (7)
Si1—O2B	1.629 (7)	C31B—C32B	1.495 (7)
Si1—O2A	1.633 (4)	C31B—H31C	0.9900
Si1—O3A	1.635 (5)	C31B—H31D	0.9900
Si1—O3B	1.644 (6)	C32B—H32D	0.9800
O1—C11B	1.431 (7)	C32B—H32E	0.9800
O1—C11A	1.441 (4)	C32B—H32F	0.9800
C11A—C12A	1.500 (5)	N1—C6^i^	1.517 (3)
C11A—H11A	0.9900	N1—C5^i^	1.524 (3)
			
N1—C1—C2	116.77 (19)	H12A—C12A—H12B	109.5
N1—C1—H1A	108.1	C11A—C12A—H12C	109.5
C2—C1—H1A	108.1	H12A—C12A—H12C	109.5
N1—C1—H1B	108.1	H12B—C12A—H12C	109.5
C2—C1—H1B	108.1	C21A—O2A—Si1	126.0 (5)
H1A—C1—H1B	107.3	O2A—C21A—C22A	108.5 (5)
N2—C2—C1	115.2 (2)	O2A—C21A—H21A	110.0
N2—C2—H2A	108.5	C22A—C21A—H21A	110.0
C1—C2—H2A	108.5	O2A—C21A—H21B	110.0
N2—C2—H2B	108.5	C22A—C21A—H21B	110.0
C1—C2—H2B	108.5	H21A—C21A—H21B	108.4
H2A—C2—H2B	107.5	C21A—C22A—H22A	109.5
N2—C3—C4	108.1 (2)	C21A—C22A—H22B	109.5
N2—C3—H3A	110.1	H22A—C22A—H22B	109.5
C4—C3—H3A	110.1	C21A—C22A—H22C	109.5
N2—C3—H3B	110.1	H22A—C22A—H22C	109.5
C4—C3—H3B	110.1	H22B—C22A—H22C	109.5
H3A—C3—H3B	108.4	C31A—O3A—Si1	117.6 (6)
C5—C4—C3	112.9 (2)	O3A—C31A—C32A	109.8 (6)
C5—C4—H4A	109.0	O3A—C31A—H31A	109.7
C3—C4—H4A	109.0	C32A—C31A—H31A	109.7
C5—C4—H4B	109.0	O3A—C31A—H31B	109.7
C3—C4—H4B	109.0	C32A—C31A—H31B	109.7
H4A—C4—H4B	107.8	H31A—C31A—H31B	108.2
C4—C5—N1^i^	112.17 (19)	C31A—C32A—H32A	109.5
C4—C5—H5A	109.2	C31A—C32A—H32B	109.5
N1^i^—C5—H5A	109.2	H32A—C32A—H32B	109.5
C4—C5—H5B	109.2	C31A—C32A—H32C	109.5
N1^i^—C5—H5B	109.2	H32A—C32A—H32C	109.5
H5A—C5—H5B	107.9	H32B—C32A—H32C	109.5
N2—C6—N1^i^	108.71 (17)	O1—C11B—C12B	104.5 (9)
N2—C6—H6A	109.9	O1—C11B—H11C	110.9
N1^i^—C6—H6A	109.9	C12B—C11B—H11C	110.9
N2—C6—H6B	109.9	O1—C11B—H11D	110.9
N1^i^—C6—H6B	109.9	C12B—C11B—H11D	110.9
H6A—C6—H6B	108.3	H11C—C11B—H11D	108.9
C8—C7—N1	116.57 (19)	C11B—C12B—H12D	109.5
C8—C7—H7A	108.1	C11B—C12B—H12E	109.5
N1—C7—H7A	108.1	H12D—C12B—H12E	109.5
C8—C7—H7B	108.1	C11B—C12B—H12F	109.5
N1—C7—H7B	108.1	H12D—C12B—H12F	109.5
H7A—C7—H7B	107.3	H12E—C12B—H12F	109.5
C7—C8—C9	108.4 (2)	C21B—O2B—Si1	116.6 (9)
C7—C8—H8A	110.0	O2B—C21B—C22B	110.2 (11)
C9—C8—H8A	110.0	O2B—C21B—H21C	109.6
C7—C8—H8B	110.0	C22B—C21B—H21C	109.6
C9—C8—H8B	110.0	O2B—C21B—H21D	109.6
H8A—C8—H8B	108.4	C22B—C21B—H21D	109.6
C8—C9—Si1	114.03 (18)	H21C—C21B—H21D	108.1
C8—C9—H9A	108.7	C21B—C22B—H22D	109.5
Si1—C9—H9A	108.7	C21B—C22B—H22E	109.5
C8—C9—H9B	108.7	H22D—C22B—H22E	109.5
Si1—C9—H9B	108.7	C21B—C22B—H22F	109.5
H9A—C9—H9B	107.6	H22D—C22B—H22F	109.5
O1—Si1—O2B	117.9 (5)	H22E—C22B—H22F	109.5
O1—Si1—O2A	106.10 (19)	C31B—O3B—Si1	134.5 (8)
O1—Si1—O3A	103.2 (3)	O3B—C31B—C32B	110.4 (11)
O2B—Si1—O3A	103.0 (8)	O3B—C31B—H31C	109.6
O2A—Si1—O3A	112.9 (5)	C32B—C31B—H31C	109.6
O1—Si1—O3B	109.3 (5)	O3B—C31B—H31D	109.6
O2B—Si1—O3B	108.8 (9)	C32B—C31B—H31D	109.6
O2A—Si1—O3B	120.3 (7)	H31C—C31B—H31D	108.1
O1—Si1—C9	111.97 (11)	C31B—C32B—H32D	109.5
O2B—Si1—C9	103.9 (6)	C31B—C32B—H32E	109.5
O2A—Si1—C9	105.3 (3)	H32D—C32B—H32E	109.5
O3A—Si1—C9	117.0 (3)	C31B—C32B—H32F	109.5
O3B—Si1—C9	103.9 (4)	H32D—C32B—H32F	109.5
C11B—O1—C11A	53.1 (5)	H32E—C32B—H32F	109.5
C11B—O1—Si1	136.6 (7)	C1—N1—C6^i^	113.09 (17)
C11A—O1—Si1	126.6 (2)	C1—N1—C7	112.57 (18)
O1—C11A—C12A	108.8 (3)	C6^i^—N1—C7	105.79 (17)
O1—C11A—H11A	109.9	C1—N1—C5^i^	108.91 (17)
C12A—C11A—H11A	109.9	C6^i^—N1—C5^i^	107.02 (17)
O1—C11A—H11B	109.9	C7—N1—C5^i^	109.24 (18)
C12A—C11A—H11B	109.9	C6—N2—C2	114.02 (18)
H11A—C11A—H11B	108.3	C6—N2—C3	108.95 (19)
C11A—C12A—H12A	109.5	C2—N2—C3	111.64 (19)
C11A—C12A—H12B	109.5		
			
N1—C1—C2—N2	−67.5 (3)	O2A—Si1—O3A—C31A	−64.5 (8)
N2—C3—C4—C5	−52.3 (3)	O3B—Si1—O3A—C31A	62 (4)
C3—C4—C5—N1^i^	48.6 (3)	C9—Si1—O3A—C31A	57.9 (9)
N1—C7—C8—C9	−170.30 (19)	Si1—O3A—C31A—C32A	−112.4 (8)
C7—C8—C9—Si1	−175.74 (16)	C11A—O1—C11B—C12B	−9.4 (8)
C8—C9—Si1—O1	−46.8 (2)	Si1—O1—C11B—C12B	−116.4 (10)
C8—C9—Si1—O2B	−175.2 (6)	O1—Si1—O2B—C21B	51.5 (14)
C8—C9—Si1—O2A	−161.7 (2)	O2A—Si1—O2B—C21B	79 (4)
C8—C9—Si1—O3A	72.0 (5)	O3A—Si1—O2B—C21B	−61.4 (13)
C8—C9—Si1—O3B	71.0 (6)	O3B—Si1—O2B—C21B	−73.6 (13)
O2B—Si1—O1—C11B	−70.0 (11)	C9—Si1—O2B—C21B	176.1 (11)
O2A—Si1—O1—C11B	−76.2 (9)	Si1—O2B—C21B—C22B	−162.0 (13)
O3A—Si1—O1—C11B	42.7 (9)	O1—Si1—O3B—C31B	154.0 (16)
O3B—Si1—O1—C11B	54.8 (10)	O2B—Si1—O3B—C31B	−76.0 (19)
C9—Si1—O1—C11B	169.4 (8)	O2A—Si1—O3B—C31B	−83.0 (18)
O2B—Si1—O1—C11A	−142.4 (8)	O3A—Si1—O3B—C31B	−142 (6)
O2A—Si1—O1—C11A	−148.7 (4)	C9—Si1—O3B—C31B	34.3 (19)
O3A—Si1—O1—C11A	−29.7 (5)	Si1—O3B—C31B—C32B	67.1 (19)
O3B—Si1—O1—C11A	−17.6 (7)	C2—C1—N1—C6^i^	60.8 (3)
C9—Si1—O1—C11A	97.0 (3)	C2—C1—N1—C7	−59.0 (3)
C11B—O1—C11A—C12A	19.5 (9)	C2—C1—N1—C5^i^	179.6 (2)
Si1—O1—C11A—C12A	144.5 (3)	C8—C7—N1—C1	−49.9 (3)
O1—Si1—O2A—C21A	58.1 (6)	C8—C7—N1—C6^i^	−173.89 (19)
O2B—Si1—O2A—C21A	−97 (4)	C8—C7—N1—C5^i^	71.2 (3)
O3A—Si1—O2A—C21A	−54.2 (6)	N1^i^—C6—N2—C2	162.99 (18)
O3B—Si1—O2A—C21A	−66.3 (7)	N1^i^—C6—N2—C3	−71.6 (2)
C9—Si1—O2A—C21A	177.0 (5)	C1—C2—N2—C6	−60.5 (3)
Si1—O2A—C21A—C22A	141.1 (5)	C1—C2—N2—C3	175.5 (2)
O1—Si1—O3A—C31A	−178.7 (7)	C4—C3—N2—C6	63.8 (3)
O2B—Si1—O3A—C31A	−55.4 (9)	C4—C3—N2—C2	−169.4 (2)

Symmetry codes: (i) −*x*+1, −*y*, −*z*.
